# Accelerated Search for BaTiO_3_‐Based Ceramics with Large Energy Storage at Low Fields Using Machine Learning and Experimental Design

**DOI:** 10.1002/advs.201901395

**Published:** 2019-09-02

**Authors:** Ruihao Yuan, Yuan Tian, Dezhen Xue, Deqing Xue, Yumei Zhou, Xiangdong Ding, Jun Sun, Turab Lookman

**Affiliations:** ^1^ State Key Laboratory for Mechanical Behavior of Materials Xi'an Jiaotong University Xi'an 710049 China; ^2^ Theoretical Division Los Alamos National Laboratory Los Alamos NM 87545 USA

**Keywords:** Bayesian optimization, ceramics, energy storage, machine learning, optimal experimental design

## Abstract

The problem that is considered is that of maximizing the energy storage density of Pb‐free BaTiO_3_‐based dielectrics at low electric fields. It is demonstrated that how varying the size of the combinatorial search space influences the efficiency of material discovery by comparing the performance of two machine learning based approaches where different levels of physical insights are involved. It is started with physics intuition to provide guiding principles to find better performers lying in the crossover region in the composition–temperature phase diagram between the ferroelectric phase and relaxor ferroelectric phase. Such an approach is limiting for multidopant solid solutions and motivates the use of two data‐driven machine learning and design strategies with a feedback loop to experiments. Strategy I considers learning and property prediction on all the compounds, and strategy II learns to preselect compounds in the crossover region on which prediction is carried out. By performing only two active learning loops via strategy II, the compound (Ba_0.86_Ca_0.14_)(Ti_0.79_Zr_0.11_Hf_0.10_)O_3_ is synthesized with the largest energy storage density ≈73 mJ cm^−3^ at a field of 20 kV cm^−1^, and an insight into the relative performance of the strategies using varying levels of knowledge is provided.

## Graphical abstract

**Discovery of new materials with targeted properties** is often a “needle in the haystack” problem. The size of the “haystack” can be effectively narrowed down by domain expert knowledge, leaving only potential materials with better properties. The searching process by data‐driven machine learning and an optimization strategy within such a preselected “haystack” is further accelerated.

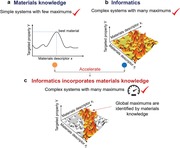

## 1 Introduction

A key challenge in developing better materials is the size of the space to be searched for the optimal chemistries and/or processing conditions. The number of experimentally synthesized compounds available to learn from is often a tiny fraction of this total allowed space and this “needle in a haystack” problem requires efficient means of sampling the space to hone in rapidly on regions of potential interest.^1^, ^2^, ^3^ We have customarily used our intuition and physics‐based understanding to guide the search for materials with better response.^4^, ^5^ For example, the susceptibility of a material can be significantly enhanced in the proximity to a phase transition region because the instabilities in the order parameters are most acute there.^6^, ^7^ However, this kind of search is still limited especially in complex systems because of the large search space associated with varying chemistry, processing conditions and microstructures, as well as the resultant size of the search space.

There has recently been much interest in employing machine learning (ML) and optimization methods to guide experimental synthesis to find materials with targeted properties.^8^, ^9^, ^10^, ^11^, ^12^, ^13^, ^14^, ^15^, ^16^, ^17^, ^18^ The state‐of‐art in this field is to use an iterative approach, which is largely data‐driven, starting from a set of features or material descriptors based on material knowledge to construct a surrogate model learned from data.^19^, ^20^ Ideas and methods from decision theory and experimental design then provide the means to make optimal decisions of the experiments or materials to test next.^19^, ^20^, ^21^, ^22^ The strategy has been quite successful in finding alloys and ceramics with enhanced properties.^11^, ^12^, ^13^, ^14^, ^23^, ^24^ Even though Bayesian based algorithms using utility functions, such as expected improvement to maximize or minimize the objective or property, have proved especially successful in finding compounds with desired properties, the search space is often too large and leads to excessive exploration. This is a reflection of a multidimensional search space, and what is needed is to isolate regions in this space containing a relatively large number of extrema so that the probability of finding a compound with the desired targeted response is much higher than in the original search space. The physics based understanding of the problem can certainly help by effectively narrowing down the search space. To this end, we compare two machine learning based strategies using varying levels of physics input to control the search space and difficulty of the task to be performed.

We will focus on the problem of finding compounds with better dielectric energy storage density from as few experiments as possible from a given training data in the easily synthesized BaTiO_3_‐based ceramics. Dielectrics capable of storing and releasing charges are the essential elements in modern electronics and electrical applications such as hybrid electric vehicles, portable electronic devices as well as power pulse devices because of their high power densities owing to the much faster charge–discharge speed than the electrochemical counterparts.^25^, ^26^, ^27^, ^28^ Studies of energy storage in devices have largely focused on performance at high electric fields aimed at increasing breakdown dielectric strengths.^25^ For example, polymer‐based dielectrics and thin films have attracted much interest in achieving superior energy storage density at very high electric fields where the break down field *E*_b_ can reach 6000 kV cm^−1^ or more.^29^, ^30^ The increase in applied electric field, however, is challenging to the supporting insulation system in the device. It often limits the applications using miniaturized components as well as portable or wearable electronic devices demanding high level of integration. Therefore, it is desirable to search for energy storage materials at relatively small electric field strengths with manageable larger energy storage density. For example, Gao et al. synthesized Ba(Ti_0.895_Sn_0.105_)O_3_ with an energy storage density of ≈55 mJ cm^−3^ at 20 kV cm^−1^ based on operating in the region of tricritical behavior in the phase diagram. This exceeds most of the ferroelectric materials at the same field strength.^31^, ^32^ In the present study, we introduce several dopants into the BaTiO_3_ prototype to search for solid solutions with enhanced energy storage density at a field of 20 kV cm^−1^. The Ba^2+^ is substituted by Ca^2+^, Sr^2+^, and the Ti^4+^ by Zr^4+^, Sn^4+^, and Hf^4+^, which gives rise to a vast search space of almost 9 million candidate choices as the concentration of each cation can be controlled to 0.01 in the synthesis process. It is too large for an Edisonian‐based approach. Hence, our focus will be on finding solid‐solutions with large dielectric energy storage density from this ≈9 million space of possible compounds using machine learning to guide experiments.

We will address this problem in two ways, (A) we will use a physics approach and consider the phase diagram of the BaTiO_3_ family from a different perspective than considered previously, namely, we will define a crossover region in the composition–temperature phase diagram between the ferroelectric phase and relaxor ferroelectric phase. Our hypothesis is that the energy storage density is enhanced in this crossover region in the composition‐temperature diagram and use this as a design principle in (B). We will provide experimental results on BaTi_1−*x*_Zr_*x*_O_3_, BaTi_1−*x*_Hf_*x*_O_3_, and BaTi_1−*x*_Sn_*x*_O_3_, to validate this hypothesis. (B) We will use two data‐driven ML strategies. Strategy I requires us to search across a relatively large search space exceeding 9 million possible compounds. We will use regression to build a surrogate model and then perform experimental design iteratively to make the best selections for synthesis. Strategy II will constrain the initial search space by using input from (A) to search only within a subclass of compounds in the crossover region to increase the probability of finding those compounds likely to lead to enhanced properties. We will do this by first using classification learning to predict compounds belonging to this subclass only, and then perform regression and experimental design on these compounds to predict the one to consider next for synthesis. We compare in this work the performance of the different strategies. We will show that formulating the materials design question appropriately, as in strategy II, not only minimizes the number of experiments required (two) to find compounds with better energy storage density than the best in our training data, but also leads to compounds with even better energy storage density compared to the ML strategy I or the approach of (A). The best compound (Ba_0.86_Ca_0.14_)(Ti_0.79_Zr_0.11_Hf_0.10_)O_3_ we have found in this work shows superior energy storage density ≈73 mJ cm^−3^ at a field of 20 kV cm^−1^, particularly accompanied with excellent energy storage efficiency (≈90%). This exceeds the performance of most of the ferroelectric materials known today at the same field strength.

## 2 Results

**Figure 1:**
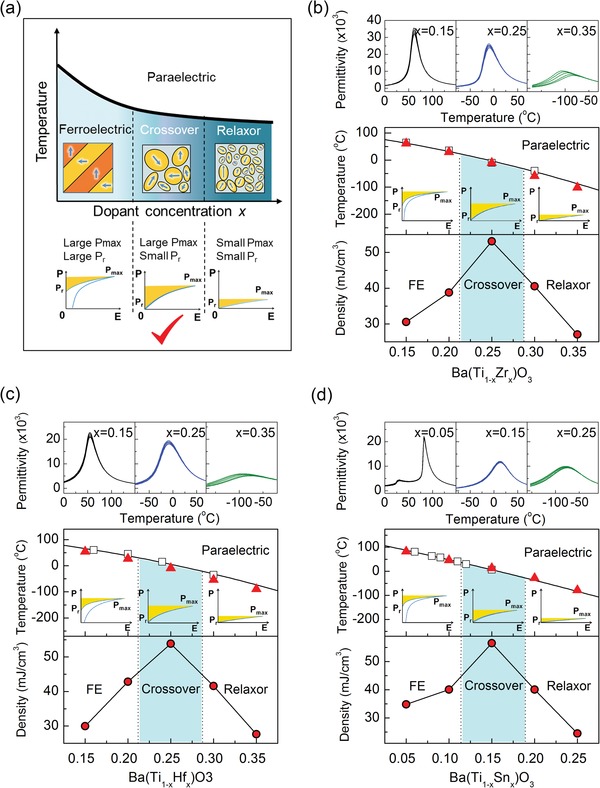
Schema shows *U*_re_ of different regions and validation in three simple systems. a) Schema from physical perspective shows that compounds located in the crossover region have higher *U*_re_, b–d) three simple systems, BaTi_1−*x*_Zr_*x*_O_3_, BaTi_1−*x*_Hf_*x*_O_3_, and BaTi_1−*x*_Sn_*x*_O_3_ show compounds in the crossover region indeed have higher *U*_re_ compared to relaxors and ferroelctrics, as highlighted by the shaded area. Moreover, our composition‐temperature phase diagrams (red triangles) show a good agreement compared to studies in the literatures (squares), frequency dependence of permittivity of typical samples are also presented.

*Physics Insights from the Phase Diagram*: In general, the energy storage density *U*_re_ of ferroelectrics (shaded area of the polarization–electric field curves in **Figure** **1**a) is given by $U_{\mathrm{re}}=\int_{P_{r}}^{P_{\max}}EdP$, where *P*_max_ is the maximum polarization and *P*_r_ is the remnant polarization.^33^ Therefore, *U*_re_ is strongly dependent on *P*_r_, *P*_max_, and *E*, where the maximum of *E* is limited by the dielectric breakdown strength (*E*_b_). To improve *U*_re_, a low *P*_r_ and a high *P*_max_ can potentially be realized by chemically modification and designed by utilizing the phase diagram of ferroelectrics

A schematic phase diagram showing how the *U*_re_ is influenced by varying the dopant content is shown in Figure 1a. As it possesses several symmetry allowed degenerate polarization states separated by large energy barriers, a long‐range ordered ferroelectric exhibits a square polarization–electric field (*P–E*) loop.^34^ Thus even though the *P*_max_ is large, a large *P*_r_ is also retained, leading to low *U*_re_ and efficiency. Inserting more point defects into a normal ferroelectric can generate an abnormal disordered polarization state, usually referred to as the relaxor ferroelectric state.^35^, ^36^, ^37^ In contrast to the large ordered domains of micrometer size in normal ferroelectrics, the microstructure of relaxors is characterized by polar nanoregions (PNR) embedded in a paraelectric matrix.^35^ The state can be “kinetically frozen” with only short‐range order and its response to electric stimuli is similar to linear dielectrics, that is, small *P*_r_ and *P*_max_, leading to a low *U*_re_.^38^, ^39^ However, in the compositional crossover region between normal ferroelectrics and relaxor ferroelectrics, the state is still disordered but not fully frozen. This affords an opportunity to be induced into long‐range ordered state with large *P*_max_ and to recover the initial disordered polarization state on removal of the external stimuli (a small *P*_r_).^38^, ^39^ Thus, the compositions in the crossover region potentially possess a fairly large *P*_max_ and small *P*_r_. Therefore our design recipe, after building a phase diagram containing both normal and relaxor ferroelectrics, is to select compositions in the crossover region where the *U*_re_ is expected to be optimized.

Accordingly, we synthesized and established phase diagrams for three different systems, BaTi_1−*x*_Zr_*x*_O_3_, BaTi_1−*x*_Hf_*x*_O_3_, and BaTi_1−*x*_Sn_*x*_O_3_. These systems are representative ferroelectrics and have been intensely investigated previously. The top panels of Figure 1b–d show typical permittivity versus temperature (ε–*T*) curves at various frequencies for the three systems, respectively. A sharp transition permittivity peak without frequency dispersion characterizes the normal ferroelectric at low dopant concentration and a smeared permittivity peak with frequency dispersion is the principal feature of the relaxor ferroelectric at high dopant concentration. The further identification of the phase transition type using modified Curie–Weiss law can be seen in Figure S1 (Supporting Information). The middle panels of Figure 1b–d give the composition–temperature phase diagrams, which agree well with those reported in the literatures,^40^, ^41^, ^42^ with three distinct composition regions for normal ferroelectrics, crossover, and relaxor ferroelectrics. The concentration dependence of the *U*_re_ calculated from *P–E* curves (Figure S1, Supporting Information) is plotted in the bottom panels of Figure 1b–d corresponding to each phase diagram. A peak value indeed appears in all three systems at the crossover region.

The *U*_re_ obtained in the above simple systems (≈55 mJ cm^−3^ at the crossover composition) is not particularly large, and chemical modification to both A site and B site of the perovskite structure should be performed to further optimize the *U*_re_. Although this design approach with insights from the phase diagram works well for the above systems, it is rather difficult to apply this recipe to multicomponent compounds, for example, (Ba_1−*x*−*y*_Ca_*x*_Sr_*y*_)(Ti_1−*u*−*v*−*w*_Zr_*u*_Sn_*v*_Hf_*w*_)O_3_ as the combination of different ions results in numerous possible pseudobinary phase diagrams. We constrain the mole fractions *x*, *y*, *u*, *v*, and *w* by 1 − *x* − *y* ≥ 0.6, *x* ≤ 0.4, *y* ≤ 0.3, 1 − *u* − *v* − *w* ≥ 0.6, *u* ≤ 0.3, *v* ≤ 0.3, and *w* ≤ 0.3, respectively. The bounds are set by considering the solubility of the dopants and ensures the transition temperature is not too low to be detected. With an unexplored space of ≈9 million possible compounds, we will use two ML and global optimization strategies within an iterative feedback loop to search for materials with enhanced *U*_re_.

**Figure 2:**
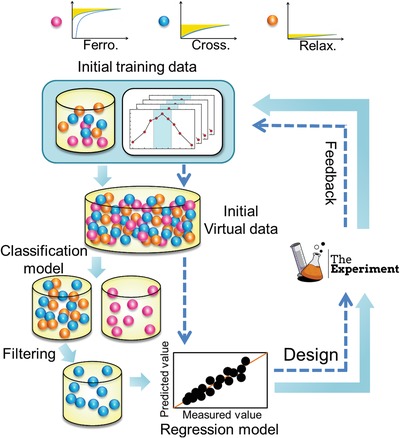
Two closed feedback loops. Inner loop with dashed arrows includes only regression ML model used in strategy I. Outer loop combines classification with regression ML models and will be used in strategy II. In each iteration, experimental results were accumulated into the training data and new models were trained and used for the next iteration.

*Strategy I: Searching from All Compounds in Phase Diagram*: We employed a purely data‐driven strategy with a training data set of 182 compounds and a search space of ≈9 million possible compounds. The training data set was obtained from the same lab using traditional solid‐solid reaction. The data file can be found in Supporting Information. The inner loop in **Figure** **2** schematically shows the feedback loop, including key ingredients: i) a training data ≈0.002% of the whole search space, each with the targeted *U*_re_ measured; ii) a ML model that uses the training data to learn the relationship between features, *x*_*i*_ and property, *U*_re_, i.e., *U*_re_ = *f*(*x*_*i*_); iii) the trained model is applied to the search space of ≈9 million unexplored compositions to predict *U*_re_ with associated model uncertainty; iv) utility function used to maximize the expected utility aids in the selection of the next candidate material; and v) feedback from experiments with subsequent improvement of the ML model.

Each compound is described in terms of features, *x*_*i*_, which include four different attributes. The change of Curie temperature due to the addition of dopants to the BaTiO_3_ matrix is important, which is abbreviated as NCT. If the doped element decreases the Curie temperature, NCT is assigned a value of −1; if the doped element increases the Curie temperature, NCT is assigned +1; and if the doped element has no effect on the Curie temperature, NCT is assigned a value of 0. For example, the addition of Zr element into the BaTiO_3_ matrix will decrease the Curie temperature, thus NCT for Zr is −1. The “tolerance factor” (*t*), the ionic displacement of B‐site elements (DB), and the ratio of the element polarizability of A‐site and B‐site elements (*P*). The feature for a given compound is then calculated as the weighted mean using the mole fraction of each element. The detailed definition and selection of features are given in Section S2 (Supporting Information). The property *U*_re_ was obtained by integrating the *P–E* loop measured under an electric field of 20 kV cm^−1^ for each sample in the training data.

**Figure 3:**
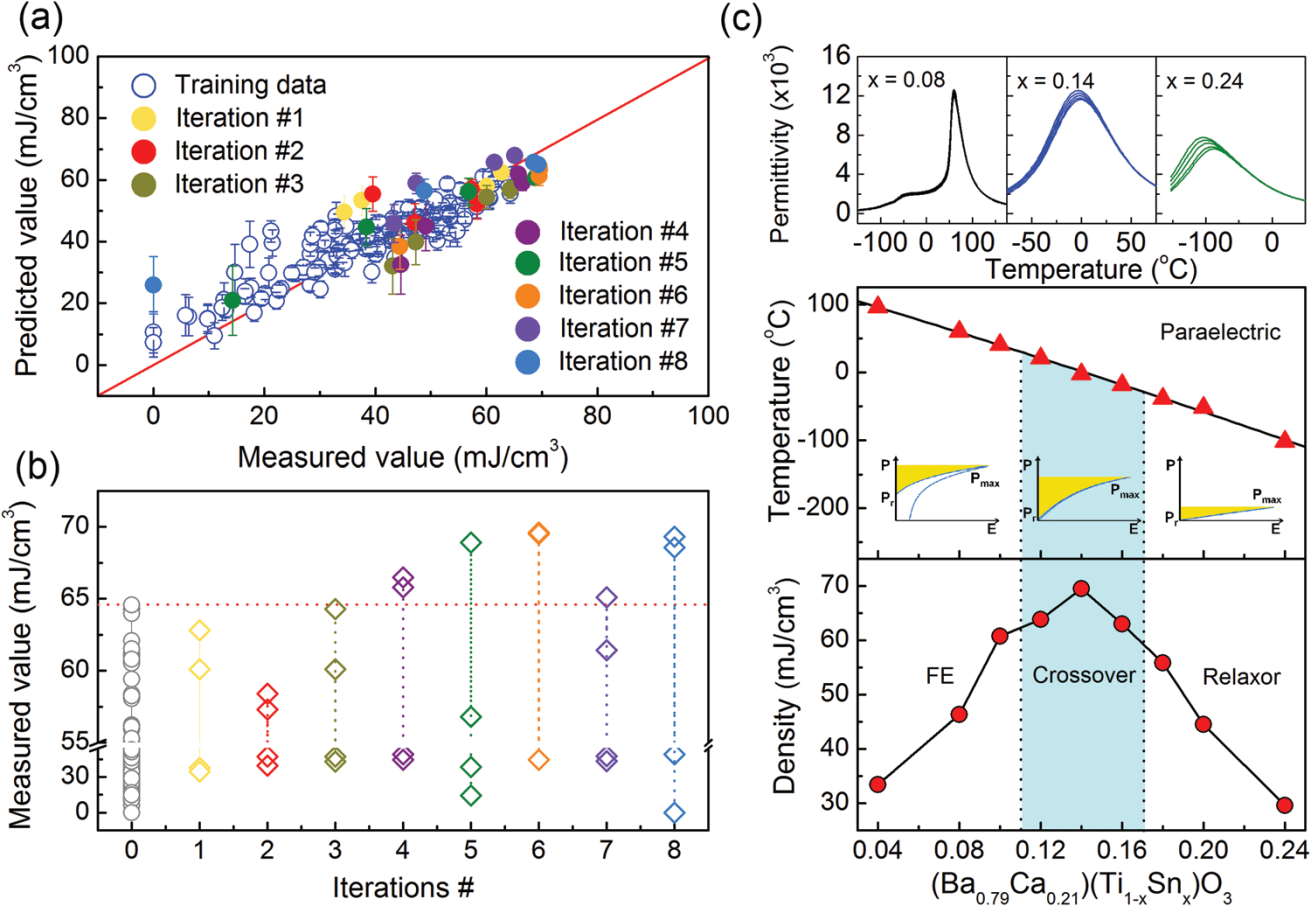
The regression model performance and the new compounds found in strategy I. a) Predicted values obtained from ML model as a function of measured values, error bar is from bootstrap sampling. b) Measured values as a function of iteration #, red line represents the best *U*_re_ in the training data, best material is found in the 6th iteration. c) Typical permittivity spectra from which the composition–temperature phase diagram of (Ba_0.79_Ca_0.21_)(Ti_1−*x*_Sn_*x*_)O_3_ is established. Crossover region is shadowed and shows better *U*_re_.

We built and compared 4 different ML models to estimate *U*_re_ = *f*(NCT, *t*, DB, *P*) and selected the support vector machine with a radial‐based kernel function (SVR.rbf) based on their mean squared and cross‐validation errors. The details of model selection are given in Section S3 (Supporting Information). The performance of this SVR.rbf model comparing the predicted mean values and experimental measured values is plotted in **Figure** **3**a. We used cross‐validation to find the hyperparameters for a robust SVR.rbf model, balancing the trade‐off between overfitting and under‐fitting. The mean values μ of the predicted property together with the associated standard deviation σ are obtained by the “bootstrap” method of statistics by randomly sampling 1000 times with repeats from the training data of 182 compounds to build 1000 models. Thus the SVR.rbf model predicts the value of *U*_re_ of unexplored compounds and the associated uncertainties.

Choosing proper candidates for next experiment is then crucial to efficiently search for the materials with better *U*_re_. We used efficient global optimization (EGO), which balances the exploration and exploitation aspects of the search to select next candidates.^13^, ^22^, ^43^ EGO evaluates the “expected improvement” (EI), a measure of the possible improvement with respect to the best performer in the training data. Also, $\mathrm{EI}=E[\max(y-\mu^{*},0)]=\int_{\mu^{*}}^{\infty }\left(y-\mu^{*})P(y|x\prime \right)\;dy=\sigma [\phi (z)+z\Phi (z)]$, where *y* − μ* is the possible improvement for certain *y* or energy density, *P*(*y*∣*x*′) is the distribution of the predicted *y* and is assumed to be normal, and *z* = (μ − μ*)/σ, where μ* is the maximum value of the energy density (*U*_re_) in the training data, μ and σ are the predicted value of *U*_re_ and the associated uncertainty, respectively. The φ(*z*) and Φ(*z*) are the standard normal density and distribution functions, respectively. Maximizing EI provides an optimal means to balance trade‐off between exploitation (σ → 0) and exploration (σ → ∞).^22^ This selector considers both the predicted value and its associated uncertainty to choose the next experiment, in contrast to merely choosing the largest predicted value. As we can synthesize four compounds at a time, we predicted four compounds, which were synthesized and characterized and the training data augmented.

We iterated the feedback loop eight times, and the measured versus predicted *U*_re_ with uncertainties are plotted on the top of the initially trained ML model in Figure 3a with solid points. Figure 3b shows the measured *U*_re_ of newly made 32 compounds (listed in Table S2 in the Supporting Information) as a function of 8 iterations. The best *U*_re_ in each iteration decreases first and increases until the peak value shown in the 6th iteration, followed by another decrease. The behavior of the predicted values as a function of iterations can be found in Figure S9 (Supporting Information), which presents a similar tendency to that in Figure 3b. The three best compounds were obtained in the 6th iteration after synthesizing and characterizing 24 compounds. The best *U*_re_ has a value of 69.5 mJ cm^−3^, which is about 8% higher than that of the best one in the training data.

To validate the assumption that the best compound we discovered is located within the crossover region, we experimentally established a composition‐temperature pseudo‐binary phase diagram by varying the Sn^4+^ content with fixed A‐site cations in the best performing compound, (Ba_0.79_Ca_0.21_)(Ti_0.86_Sn_0.14_)O_3_. The phase diagram of (Ba_0.79_Ca_0.21_)(Ti_1−*x*_Sn_*x*_)O_3_ is shown in Figure 3c. The ε–*T* curves of various Sn^4+^ compounds (Figure S10, Supporting Information) demonstrate that the best compound from strategy I is located in the crossover region. Typical curves are shown in the top panel of Figure 3c and the crossover region is highlighted by the shadow. The *U*_re_ obtained from *P–E* curves (Figure S10, Supporting Information) is plotted as a function of Sn^4+^ content in the bottom panel. The best compound (Ba_0.79_Ca_0.21_)(Ti_0.86_Sn_0.14_)O_3_ outperforms the surrounding compositions, indicating at least one local maximum of the search space.

*Strategy II: Searching from Preselected Compounds in Crossover Region*: This strategy only consider the compounds that fall within the crossover regions. As these compounds are likely the extrema of *U*_re_, we anticipate that the design optimization will be more favorable and less exploratory in terms of number of iterations required. The strategy is schematically shown by the outer loop in Figure 2. A key difference compared to strategy I is that a binary classification model is first used to down select crossover region compounds. It is noted that a multiclass classifier to “ferroelectric”, “relaxor”, and “crossover” is an alternative way to predict the “crossover” compositions.

**Figure 4:**
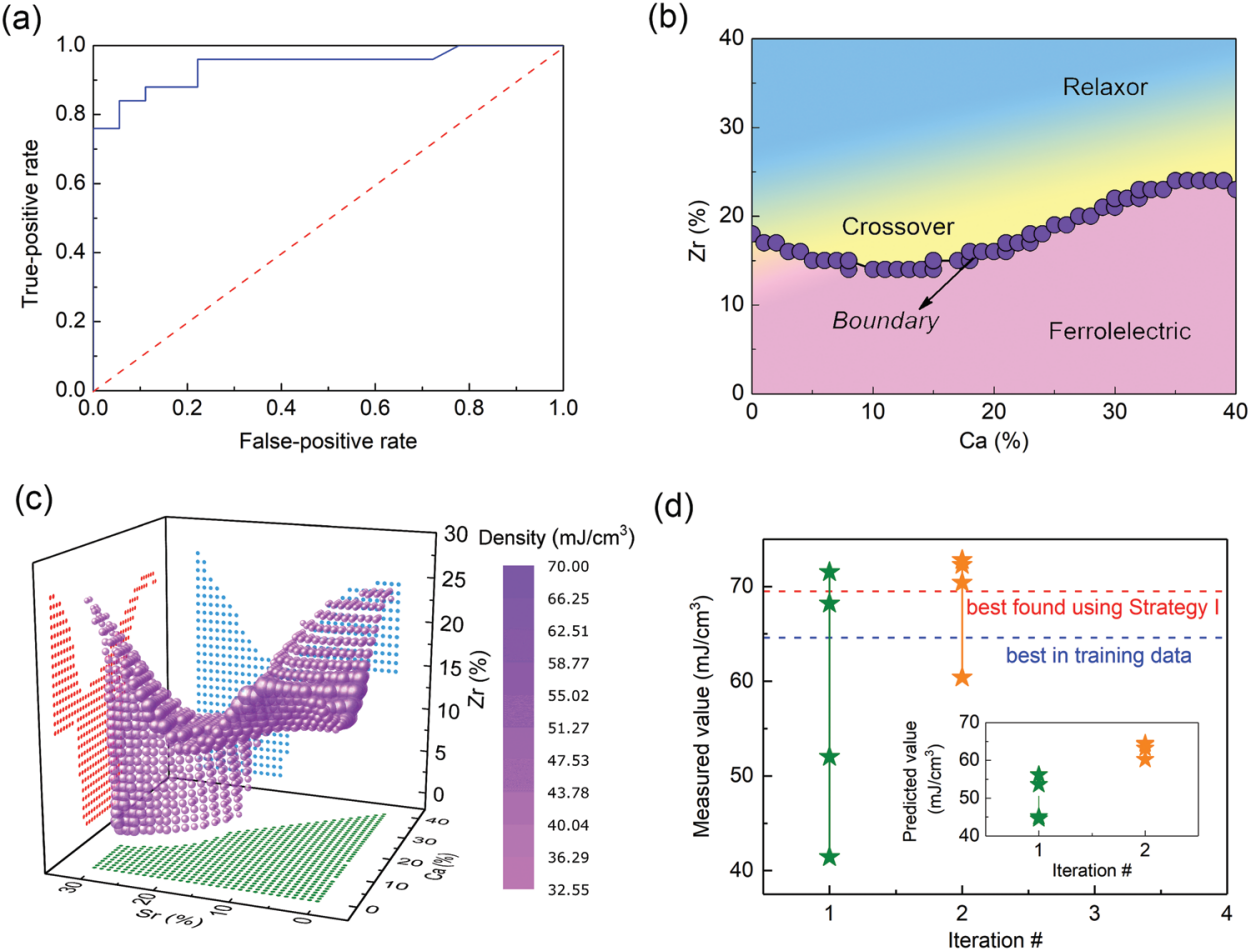
Performance of the classification model and the new 8 compounds synthesized in strategy II. a) ROC curve to validate our classifier. b) A projection of the boundary to a 2D phase diagram featured by elements Zr and Ca. c) A 3D phase diagram to show the boundary between normal ferroelectrics and relaxors (includes crossover region) predicted from classification model; above the boundary are compounds in crossover and relaxor region, below are normal ferroelectrics. d) Measured values as a function of number of iterations; inset shows that the predicted values change with iterations. Red and blue dashed lines indicate the best *U*_re_ found in strategy I and in training data, respectively.

The training data for classification includes 183 compounds labeled as either ferroelectric or relaxor (Supporting Information). The training data thus has 56 samples labeled relaxor and 127 samples labeled ferroelectric. Compounds in the crossover region show a little frequency dispersion in the dielectric spectrum and therefore we labeled them as relaxors. we used the mole fraction of each ion as feature to build the classifier, which was a support vector machine with a radial‐based kernel function. We calculated the receiver operating characteristic (ROC) curve that uses the probability of prediction to evaluate the performance of a binary classification model. Every point on the line shows how the classifier performs at a given threshold.^44^ The initial labeled 183 compounds were randomly separated into a training set (140 compounds) and a test set (43 compounds). The same algorithm was used to train a model in the training set and then validated on the test set. **Figure** **4**a shows the ROC curve based on the test set, where the red dashed line represents the random guess. The area under the ROC curve is 0.94 which ensures a robust classification model. We also evaluated the performance of the classifier by considering training, cross‐validation and test errors (details can be seen in Figure S11 in the Supporting Information). The misclassification rate for each compound in the training data is also calculated using the bootstrap method and listed in Table S3 (Supporting Information).

We applied the classifier to the unexplored space of ≈9 million compounds, with each compound assigned as either ferroelectric or relaxor. The compounds on the phase boundary between ferroelectric and relaxor were determined by monitoring their nearest neighbors. For each compound in the unexplored space, if at least one nearest neighbor is labeled differently from the others, this compound is considered to be on the boundary. To clarify, Figure 4b shows a 2D phase diagram where the axes are the features monitoring the composition of Zr and Ca. The boundary (or the margin) clearly distinguishes ferroelectrics from relaxors. Given that we have a crossover region rather than just a boundary between the ferroelectrics and relaxors, we incorporate compounds within the crossover region by considering compounds up to four neighbors or steps into the relaxor region. As a 5D plot is challenging to visualize, a plot of the boundary between normal ferroelectrics and relaxors in three dimensions is shown in Figure 4c, in terms of the Zr, Ca, and Sr compositions with the relaxors above separated from the ferroelectrics below by the compounds on the boundary. The crossover region includes ≈690 000 compounds in total and is much smaller compared to the whole unexplored space of ≈9 million compounds. Most importantly, the compounds in this smaller search space have the most promising potential to show enhanced *U*_re_.

The same regression model based on 182 compounds (≈0.025% of the smaller unexplored space) trained in strategy I was employed to make predictions on this smaller search space. We again used expected improvement as the selection criterion, as shown in Figure 2. After synthesis and characterization, the experimental results were added to the training data for both classification and regression, and subsequent iteration proceeds accordingly. The outer loop in Figure 2 iterates twice and 8 new compounds were synthesized and characterized. The corresponding measured *U*_re_ (calculated from *P–E* curves in Figure S12 in the Supporting Information) are listed in **Table** **1**. Five samples show comparable or higher *U*_re_ than the best value found in strategy I (red dashed line), as shown in Figure 4d. The best compound (Ba_0.86_Ca_0.14_)(Ti_0.79_Zr_0.11_Hf_0.10_)O_3_ found in strategy II shows a *U*_re_ of ≈73 mJ cm^−3^, improved by 14% compared to the best in the training data. Inset plot indicates that predicted values versus iterations show a similar tendency to the measured values. The ε–*T* curves in Figure S13 (Supporting Information) show a degree of diffusion, confirming that all 5 new compounds are likely located in the crossover region. Thus, invoking the physics associated with the crossover region between normal and relaxor ferroelectrics has allowed us to effectively reduce the search space to significantly accelerate finding high *U*_re_ compounds.

**Table 1:** The 8 new compounds synthesized in strategy II (five of these (in bold) have a comparable or higher *U*_re_ than the best found in strategy I)

Iteration #	Ba	Ca	Sr	Ti	Zr	Sn	Hf	*U*_re_
1	0.97	0.03	0.00	0.86	0.00	0.14	0.00	52.1
1	0.61	0.09	0.30	0.90	0.10	0.00	0.00	41.4
1	0.81	0.19	0.00	0.84	0.00	0.16	0.00	**68.2**
1	0.87	0.13	0.00	0.79	0.11	0.00	0.10	**71.5**
2	0.86	0.14	0.00	0.79	0.05	0.00	0.16	**70.4**
2	0.80	0.20	0.00	0.83	0.00	0.17	0.00	60.4
2	0.87	0.13	0.00	0.79	0.10	0.00	0.11	**72.3**
2	0.86	0.14	0.00	0.79	0.11	0.00	0.10	**72.8**

## 3 Discussion and Summary

**Figure 5:**
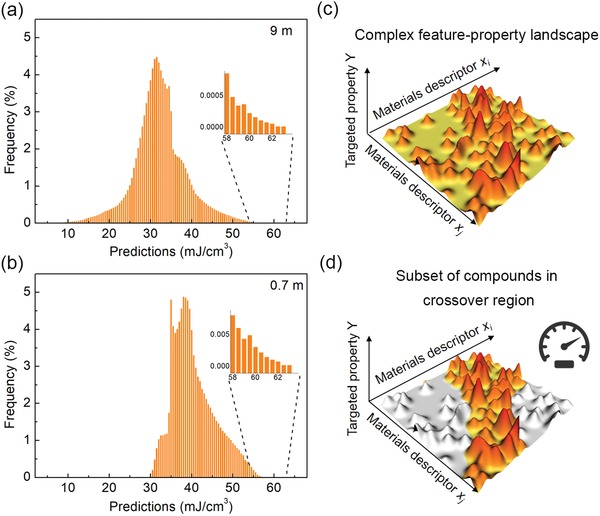
Distribution of predictions of the virtual space used in strategy I and strategy II. a) The distribution of predicted *U*_re_ of virtual space in strategy I, inset is the amplification of the part with large predicted values. b) The distribution of predicted *U*_re_ of virtual space in strategy II, inset is the amplification of the part with large predicted values. c) Schema to show a complex landscape with many local extrema. d) Major local extrema are removed from the virtual space using physical understanding, there is a greater probability of finding large *U*_re_ in this landscape.

To glean some insights into the two strategies, **Figure** **5**a shows the initial distribution of predictions of the virtual space (≈9 million) for strategy I, where the predictions vary from 5 to 63 mJ cm^−3^ with a peak located at a *U*_re_ around 30 mJ cm^−3^. The initial distribution of predictions in the virtual space for ≈0.7 million compounds in strategy II shifts to higher values, as shown in Figure 5b. All the predictions are larger than 30 mJ cm^−3^ and the range now is from 30 to 63 mJ cm^−3^, in particular, the peak is located at a *U*_re_ around 39 mJ cm^−3^. Thus, compared to strategy I that possesses many local extrema as shown in Figure 5c, strategy II effectively preselects a subset of compounds (using classification model) from the total space that are likely to be favored, as shown in Figure 5d. This will avoid excess exploration around local extrema.

In summary, we proposed a guiding principle which finds better energy storage properties in the crossover region of the composition–temperature phase diagram between the ferroelectric phase and relaxor ferroelectric phase. As a prior information of phase diagram is needed, this approach is limiting in complex multicomponent systems. We thus have compared the performance of two ML strategies to find multicomponent solid‐solutions with enhanced energy storage density, *U*_re_ in BaTiO_3_‐based ceramics using experimental data. Strategy I applied ML model and adaptive design directly to a huge virtual space of ≈9 million compounds. After 6 iterations, compositions were found that improved the targeted property by 8% compared to the best in the training data. By using physical insights from the composition‐temperature phase diagram that allowed only a subclass of compounds to be considered in the virtual space, strategy II utilized ML in the form of classification and regression models, as well as adaptive design. The search therefore becomes far more efficient as only 2 iterations were needed to obtain 5 compounds with comparable or higher *U*_re_ than the best found in strategy I. The best compound (Ba_0.86_Ca_0.14_)(Ti_0.79_Zr_0.11_Hf_0.10_)O_3_ found in the reduced search space (with 5 easy to process elements) has a *U*_re_ of ≈73 mJ cm^−3^ at 20 kV cm^−1^, 14% better than the best in the training data. The relative performance of the two strategies is revealed from a statistical perspective. We envision the proposed framework can be extended to other complex systems to accelerate the searching process of new materials.

## 4 Experimental Section

*Experimental*: (Ba_1−*x*−*y*_Ca_*x*_Sr_*y*_)(Ti_1−*u*−*v*−*w*_Zr_*u*_Sn_*v*_Hf_*w*_)O_3_ ceramics were fabricated by a conventional solid–solid reaction method with the starting materials of BaCO_3_ (99.8%), CaCO_3_ (99.9%), SrCO_3_ (99.9%), BaZrO_3_ (99.9%), SnO_2_ (99.9%), HfO_2_(99.8%), and TiO_2_ (99.6%). The calcination was performed at 1350 °C for 3 h and sintering was done at 1450 °C for 3 h in air. All the samples were synthesized under the same conditions to reduce the dependence of targeted property on processing. The sintered samples for ferroelectric and dielectric measurements were polished to obtain parallel sides and coated with silver electrodes. The polarization–electric field (*P–E*) loops were identified by a ferroelectric workstation at 10 Hz and frequency dependence of permittivity on temperature were measured using a HIOKI 3532‐50 LCR meter, all with disk‐shaped samples.

*Machine Learning Models*: Four ML regression models are employed in this study, support vector machine with a radial‐based kernel function, ridge regression with a radial‐based kernel function, gradient boosting decision tree, and random forest. The former two models use tenfolds cross‐validation to choose the best hyperparameters. In the first three models, the predictions and uncertainties are obtained from 1000 models built on 1000 resampling training data with repeats. For the random forest model, 500 000 trees are trained based on the resampling method with repeats. In terms of the classification model, support vector machine with a radial‐based kernel function is used, and leave‐one‐out cross‐validation is used to choose the best hyperparameters. The support vector machine with a radial‐based kernel function for regression and classification was implemented in the e1017 package within the RSTUDIO environment.

## Conflict of Interest

The authors declare no conflict of interest.

## Supporting information

Supporting Information
