# Supplementary material for: Accelerated Search for BaTiO3‐Based Ceramics with Large Energy Storage at Low Fields Using Machine Learning and Experimental Design
Source: Adv Sci (Weinh). 2019 Sep 2;6(21):1901395. doi: 10.1002/advs.201901395 (PMC6839636; doi:10.1002/advs.201901395)
Supplement: Supplementary file 1 — Supporting Information [file ADVS-6-1901395-s001.pdf]

Copyright WILEY-VCH Verlag GmbH & Co. KGaA, 69469 Weinheim, Germany, 2019.

Supporting Information

for *Adv. Sci.,* DOI: 10.1002/advs.201901395

Accelerated Search for $BaTiO_3$-Based Ceramics with Large Energy Storage at Low Fields Using Machine Learning and Experimental Design

Ruihao Yuan, Yuan Tian, Dezhen Xue, Deqing Xue, Yumei Zhou, Xiangdong Ding,* Jun Sun, and Turab Lookman**

Accelerated Search for $BaTiO_3$-based Ceramics with Large Energy Storage at Low Fields Using Machine Learning and Experimental Design

Ruihao Yuan,[1,2] Yuan Tian,[1] Dezhen Xue,[1,*] Deqing Xue,[1] Yumei Zhou,[1] Xiangdong Ding,[1,†] Jun Sun,[1] and Turab Lookman[2,‡]

[1]*State Key Laboratory for Mechanical Behavior of Materials, Xi'an Jiaotong University, Xi'an 710049, China.*
[2]*Theoretical Division, Los Alamos National Laboratory, Los Alamos, New Mexico 87545, USA.*

Section 1: Dielectric and ferroelectric properties of simple systems

Figures S1(a1)-(c1) show the dielectric permittivity (ε) versus temperature (T) curves at different frequencies in the $BaTi_{1-x}Zr_xO_3$, $BaTi_{1-x}Hf_xO_3$ and $BaTi_{1-x}Sn_xO_3$ system, respectively. The different colors represent various compounds in the system. As the dopant increases, the temperature window of the dielectric permittivity peak becomes wider. Moreover, there is a clear frequency dispersion once x exceeds a critical value. A modified Curie-Weiss law has been proposed to describe the diffused phase transition,

$$1/\varepsilon - 1/\varepsilon_m = (T - T_m)^\gamma / C' \tag{1}$$

where γ and C' are assumed to be constants. The parameter, γ gives information on the character of the phase transition: $\gamma = 1$ indicates a normal ferroelectric phase transition and $\gamma = 2$ represents a complete or ideal diffused phase transition. Figures S1(a2)-(c2) plot $\ln(1/\varepsilon - 1/\varepsilon_m)$ as a function of $\ln(T - T_m)$ for the compounds in each system, respectively. The γ value is determined from the slope of the fitted curves using equation 1. A similar tendency is seen in the three simple systems, *i.e.*, parameter γ increases with dopant concentration, x.

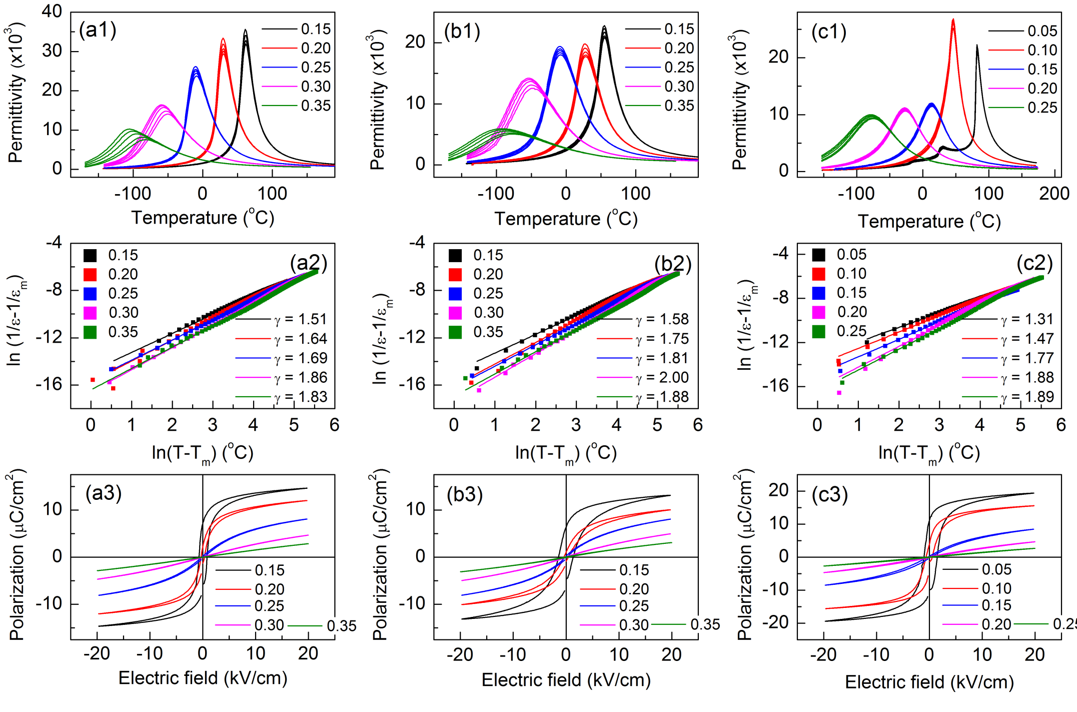

Figure S 1 | Dielectric and ferroelectric behavior of compounds in $BaTi_{1-x}Zr_xO_3$, $BaTi_{1-x}Hf_xO_3$ and $BaTi_{1-x}Sn_xO_3$. **(a1)-(c1)** Permittivity versus temperature curves in $BaTi_{1-x}Zr_xO_3$, $BaTi_{1-x}Hf_xO_3$ and $BaTi_{1-x}Sn_xO_3$, respectively. **(a2)-(c2)** Permittivity versus temperature curves fitted by modified Curie-Weiss law at high temperature, parameter γ increases with increasing dopant concentration. **(a3)-(c3)** Polarization versus electric field loops for compounds in each system.

Figures S1(a3)-(c3) show the polarization (P) versus electric field (E) loops at room temperature of compounds in each system. The P-E loop changes from fat to slim and finally to almost linear as x increase, at the same time both P_{max} and P_r decrease monotonically. The energy storage density can be calculated from these curves.

Table S 1 | The definition of the 13 selected features.

Features	Description
P	Ratio of the polarizability of A-site and B-site elements
p	Product of the polarizability of A-site and B-site elements
gr	Product of the group of A-site and B-site elements in the element period table
EVW	Ratio of the equilibrium van der Waals radii of A-site and B-site atoms
evw	Product of the equilibrium van der Waals radii of A-site and B-site atoms
DB	Ionic displacement of B-site atoms
ea	Product of the ionization energies of A-site and B-site elements
ENP	Ratio of the A-site and B-site electronegativity–Pauling scale
enp	Product of the A-site and B-site electronegativity–Pauling scale
$ENMB$	Ratio of the A-site and B-site electronegativity–Matyonov-Batsanov
$enmb$	Product of the A-site and B-site electronegativity–Matyonov-Batsanov
NCT	∈(+1, -1, 0), that captures the trend (increase, decrease or no-change) of the dependence of Curie temperature on the doping element
t	Tolerance factor calculated by Shannon's ionic radii

Section 2: Feature definition and selection

13 features are assembled based on casting a wider net of knowledge in terms of choosing features that could influence the objective. The definition of the 13 features are given in Tab. S1. The feature for a given compound can be calculated using the weighted method. For example, the feature P can be calculated by the following equation.

$$P = \frac{f^{Ba} * P^{Ba} + f^{Ca} * P^{Ca} + f^{Sr} * P^{Sr}}{f^{Ti} * P^{Ti} + f^{Zr} * P^{Zr} + f^{Sn} * P^{Sn} + f^{Hf} * P^{Hf}} \tag{2}$$

Where f^{Ba} and P^{Ba} are the mole fraction and polarizability of Ba element, respectively.

We find that the features are not particularly linearly correlated, as shown in Fig. S2.

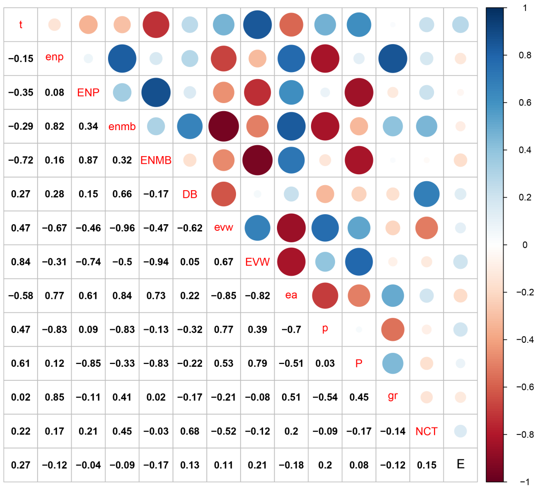

Figure S 2 | Pearson correlation matrix of the 13 assembled features, and the energy density (E).

The initial training data was divided into training data and test data randomly with a ratio 0.8/0.2. A support vector regressor with a radial-based kernel function (SVR.rbf) was built using the training data for each of the 13 features. The trained models were then applied to the test data. The mean squared error (MSE.error) calculated for both training data and test data for each feature, is shown in Fig. S3. The error bar is from the predictions of 100 repeats of the randomly divided training and test data. We can see that 'DB', 'NCT', 't' are the best performing three features. Also, the next 10 are quite similar in terms of the test error and we choose P because of its physical appeal in terms of polarization.

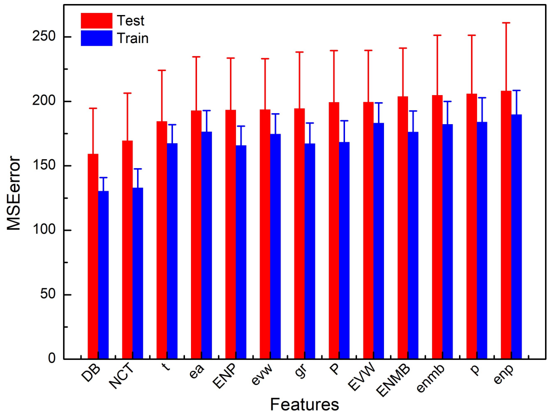

Figure S 3 | The MSE.error in the training data and test data, as a function of the features used to build the machine learning model.

We also used gradient boosting tree to rank all the 13 features by considering their relative importance. Figure S4 shows that 'NCT', 't', 'DB' are the best three, in agreement with that in Fig. S3.

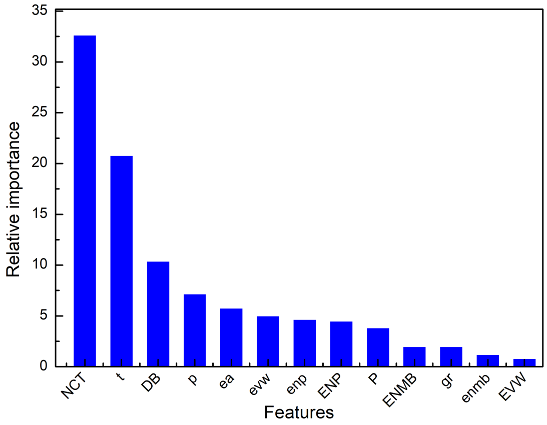

Figure S 4 | The relative importance of features ranked by gradient boosting tree.

Section 3: Regression model performance evaluate

Figure S5 shows the regression models based on the whole training data (182 compounds), (a)-(d) indicate the following four different algorithms, respectively. SVR.rbf and Random forest are the two with the best perfromance.

- SVR.rbf: support vector machine with a radial-based kernel function.
- Random forest (RF): an ensemble learning method that is trained on a multitude of decision trees and the output is the mean of predictions from individual trees.
- Gradient boosting (GB): building an additive model in a forward stage-wise fashion, in each stage a regression tree is trained to minimize the given loss function in the negative gradient direction.
- KRR: combines Ridge Regression (linear least squares with L2-norm regularization) with a radial-based kernel function.

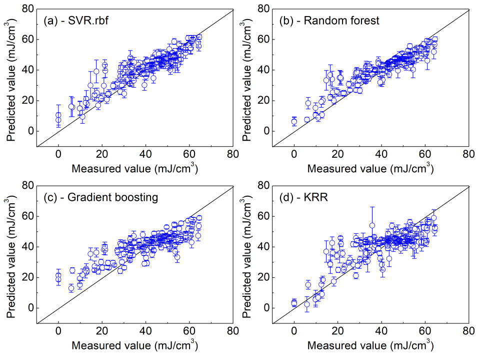

Figure S 5 | Four regression models based on the whole training data, **(a)-(d)** correspond to SVR.rbf, random forest, gradient boosting and KRR, respectively.

To evaluate the predictive performance of the regression model, we divided the whole data into two parts: a training data with 152 compounds and a test data with 30 compounds. Figures S6(a)-(d) shows the performance of various models, blue circles are the training data and red points are the test data. Red points suggest that SVR.rbf performs better than other models, especially at high energy storage density.

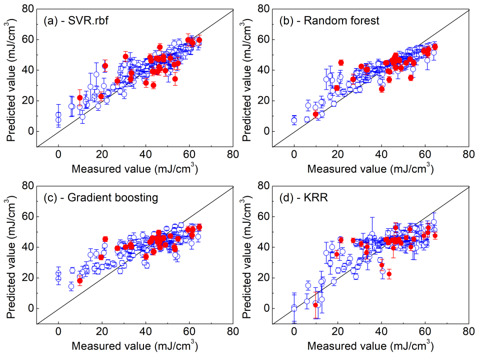

Figure S 6 | Four regression models perform on the test data. Blue circles represent 152 samples in training data and red points indicate 30 samples in the test data. **(a)-(d)** correspond to SVR.rbf, random forest, gradient boosting and KRR, respectively.

Figure S7 shows the predictive errors for several regression models. We evaluate the leave-one-out cross-validation error (CV.error) in the whole set of 182 compounds, and the mean squared error (MSE.error, based on 1000 models from bootstrap method) in the test set of 30 compounds used in Fig. S6. Considering both two errors, SVR.rbf is the better model and we use it to make predictions on the virtual data. Moreover, Fig. S8 shows that the two errors are very similar, indicating that our model is not over-fitting or under-fitting.

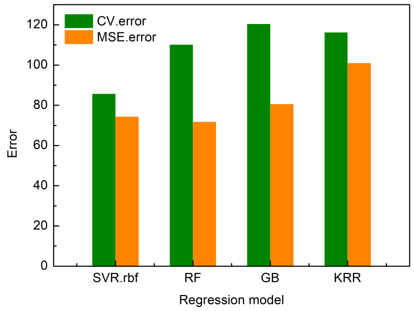

Figure S 7 | Cross validation error and mean squared error calculated for four different algorithms.

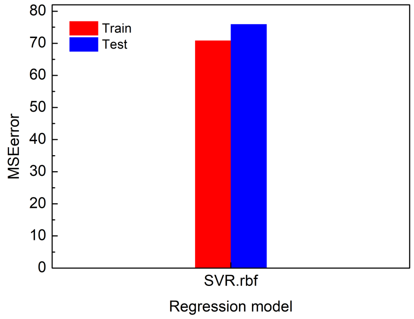

Figure S 8 | Mean squared error in the training data and test data for SVR.rbf.

Figure S9 shows the predicted energy storage density as a function of the number of iterations, which has a similar tendency with the measured values shown in Fig. 3c in the manuscript.

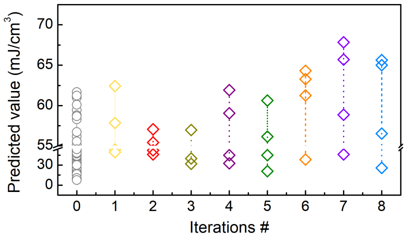

Figure S 9 | Predicted values change with the number of iterations, totally 8 iterations were carried out.

Table S 2 | 8 iterations were finished in strategy I and totally 32 new compounds are listed.

Iteration #	Ba	Ca	Sr	Ti	Zr	Sn	Hf	U_{re}
1	0.90	0.10	0.00	0.97	0.00	0.03	0.00	37.58
1	0.84	0.16	0.00	0.84	0.00	0.16	0.00	62.80
1	0.82	0.18	0.00	0.87	0.00	0.13	0.00	60.10
1	0.84	0.16	0.00	0.91	0.00	0.09	0.00	34.40
2	0.93	0.07	0.00	0.95	0.01	0.01	0.03	39.50
2	0.80	0.20	0.00	0.84	0.00	0.16	0.00	58.40
2	0.60	0.10	0.30	0.90	0.09	0.00	0.01	47.10
2	0.90	0.10	0.00	0.84	0.00	0.16	0.00	57.30
3	0.80	0.20	0.00	0.85	0.00	0.15	0.00	64.30
3	0.69	0.01	0.30	0.90	0.10	0.00	0.00	43.10
3	0.93	0.07	0.00	0.87	0.00	0.13	0.00	47.30
3	0.88	0.12	0.00	0.79	0.19	0.00	0.02	60.10
4	0.71	0.00	0.29	0.90	0.01	0.00	0.09	49.10
4	0.65	0.05	0.30	0.90	0.09	0.00	0.01	44.60
4	0.79	0.21	0.00	0.85	0.00	0.15	0.00	66.50
4	0.81	0.19	0.00	0.85	0.00	0.15	0.00	65.80
5	0.78	0.22	0.00	0.82	0.00	0.18	0.00	56.80
5	0.78	0.22	0.00	0.86	0.00	0.14	0.00	68.90
5	0.95	0.05	0.00	0.94	0.06	0.00	0.00	38.40
5	0.80	0.00	0.20	0.60	0.09	0.01	0.30	14.30
6	0.76	0.24	0.00	0.85	0.00	0.15	0.00	69.52
6	0.79	0.21	0.00	0.86	0.00	0.14	0.00	69.53
6	0.65	0.05	0.30	0.90	0.10	0.00	0.00	44.43
6	0.77	0.23	0.00	0.86	0.00	0.14	0.00	69.60
7	0.75	0.25	0.00	0.85	0.00	0.15	0.00	61.43
7	0.77	0.23	0.00	0.85	0.00	0.15	0.00	65.11
7	0.73	0.27	0.00	0.83	0.00	0.17	0.00	47.34
7	0.72	0.00	0.28	0.90	0.00	0.01	0.09	43.40
8	0.79	0.21	0.00	0.87	0.00	0.09	0.04	48.87
8	0.77	0.22	0.01	0.86	0.00	0.14	0.00	68.57
8	0.78	0.22	0.00	0.87	0.00	0.13	0.00	69.34
8	0.66	0.23	0.11	0.69	0.00	0.30	0.01	0.00

Table S2 lists all the 32 new compounds synthesized in strategy I.

Section 4: Dielectric and ferroelectric properties of $(Ba_{0.79}Ca_{0.21})(Ti_{1-x}Sn_x)O_3$ system

Figure S10(a) shows the permittivity as a function of temperature for different Sn^{4+} concentration in the $(Ba_{0.79}Ca_{0.21})(Ti_{1-x}Sn_x)O_3$ system. The plot shows a similar tendency with that in Fig. S1(a1)-(c1), the phase transition product changes from normal ferroelectrics to relaxor mediated by a "crossover region". Figure S10(b) shows how the P-E loops change with Sn^{4+} concentration.

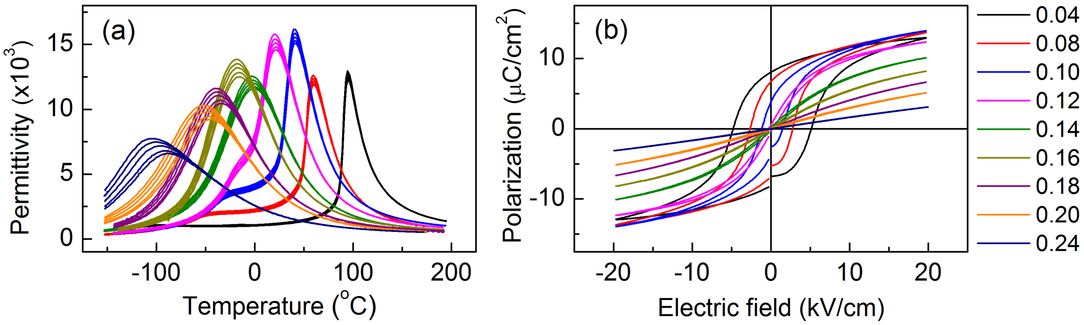

Figure S 10 | **(a)** shows the permittivity as a function of Sn^{4+} concentration, x. **(b)** shows the corresponding P-E curves.

Section 5: Classification model performance evaluate

Figure S11 shows the accuracy (error) of the classification model, we calculated both train error and predictive errors.

- Train error (Train.error) evaluates the model performance on the whole training data: all 183 labeled compounds were used to build a classifier and then applied on the same 183 compounds.
- Predictive errors include both cross-validation error (CV.error) and error on the test data (Test.error).

  CV.error: leave-one-out cross validation was used to calculate the error from the whole set of 183 compounds.

  Test.error: the whole set of 183 compounds was randomly divided into two parts: a training data with 153 compounds and a test data with 30 compounds, the classifier was trained based on 153 compounds and then applied on the other 30 compounds, from which we can calculate the Test.error.

We find that all the accuracies are higher than 0.9, demonstrating superior model performance of the classifier.

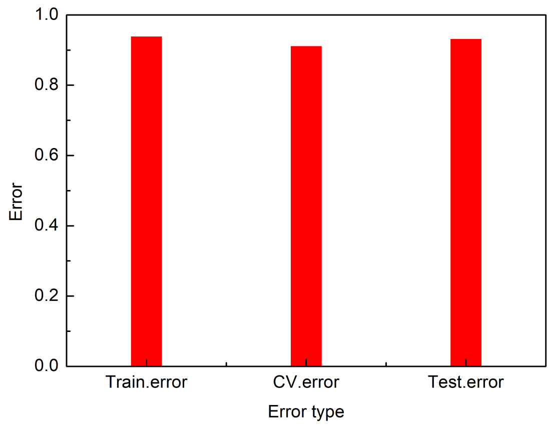

Figure S 11 | Three types of errors (accuracies) for the classifier.

Table S 3 | The 19 compounds with misclassification rate or frequency larger than 0.2 from all 183 compounds, 10 compounds have a frequency larger than 0.4 (in bold).

ID	Ba	Ca	Sr	Ti	Zr	Sn	Hf	Type	Frequency
1	0.8425	0.1575	0.00	0.8625	0.1375	0.00	0.00	Ferroelectric	0.320
2	0.95	0.00	0.05	0.80	0.20	0.00	0.00	Ferroelectric	**0.402**
3	0.90	0.00	0.10	0.80	0.20	0.00	0.00	Relaxor	0.239
4	0.84	0.16	0.00	0.868	0.00	0.132	0.00	Ferroelectric	0.396
5	0.91	0.09	0.00	0.86	0.14	0.00	0.00	Ferroelectric	0.384
6	0.91	0.09	0.00	0.89	0.00	0.11	0.00	Relaxor	**0.561**
7	0.86	0.14	0.00	0.87	0.13	0.00	0.00	Relaxor	**0.543**
8	0.76	0.08	0.16	0.85	0.06	0.09	0.00	Relaxor	0.265
9	0.85	0.15	0.00	0.87	0.06	0.07	0.00	Relaxor	0.340
10	0.95	0.05	0.00	0.89	0.00	0.11	0.00	Relaxor	**0.480**
11	0.97	0.03	0.00	0.88	0.00	0.12	0.00	Relaxor	**0.414**
12	0.83	0.04	0.13	0.75	0.06	0.19	0.00	Relaxor	0.206
13	0.85	0.15	0.00	0.83	0.00	0.17	0.00	Ferroelectric	**0.768**
14	0.83	0.17	0.00	0.85	0.00	0.15	0.00	Ferroelectric	**0.845**
15	0.82	0.18	0.00	0.87	0.00	0.13	0.00	Relaxor	**0.533**
16	0.90	0.10	0.00	0.84	0.00	0.16	0.00	Ferroelectric	**0.544**
17	0.93	0.07	0.00	0.87	0.00	0.13	0.00	Ferroelectric	**0.450**
18	1.00	0.00	0.00	0.80	0.00	0.00	0.20	Relaxor	0.260
19	0.95	0.01	0.04	0.79	0.21	0.00	0.00	Relaxor	0.239

Misclassification of compounds can be an issue and we used bootstrap sampling to obtain the misclassification rate or frequency for every compound in the whole training data. That is, for each prediction, 183 samples were selected with replacement from the original 183 compounds and based on this data, a classification model was constructed and applied to the 183 compounds. This process was repeated 1000 times to obtain the misclassification frequency for each compound. The 19 compounds with high misclassification frequency ($\geqslant 0.2$) are listed in Tab. S3. 10 compounds with much higher misclassified frequency ($\geqslant 0.4$) are presented in bold.

Section 6: Dielectric and ferroelectric properties of 8 compounds in strategy II

Figure S12(a)-(b) plot the P-E loops of 8 new compounds synthesized in strategy II.

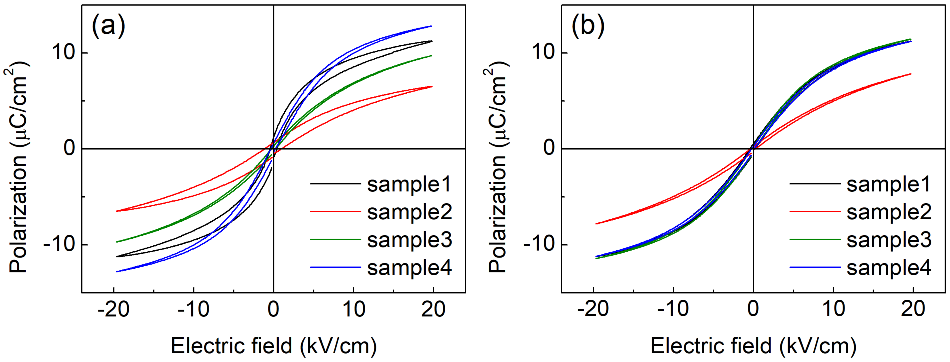

Figure S 12 | P-E loops for 8 new compounds. **(a)** represents 4 compounds in the first iteration and **(b)** represents 4 compounds in the second iteration.

Figure S13(a)-(b) plot the permittivity vs. temperature curves of 8 new compounds synthesized in strategy II.

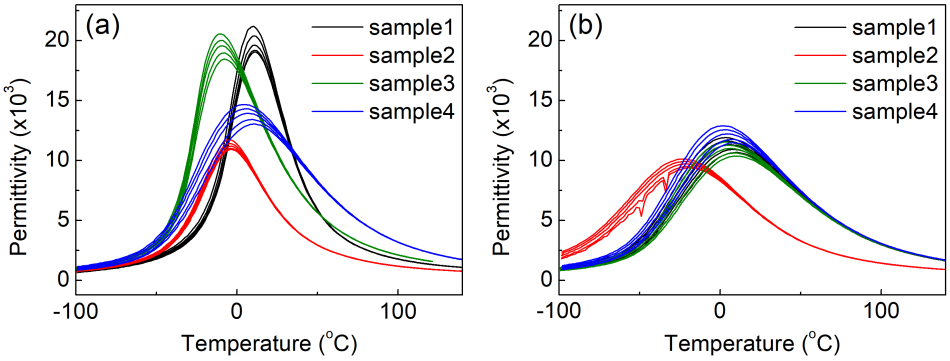

Figure S 13 | Permittivity vs. temperature curves for 8 new synthesized compounds. **(a)** represents 4 compounds in the first iteration and **(b)** represents 4 compounds in the second iteration.

Section 7: Distribution of the predictions of virtual space used in strategy II

The crossover region consists of four steps or layers as described in the main text. Figure S14(a)-(d) show the distribution of predictions for each step, respectively. Inset in each panel is the amplification of the range with high predicted values.

The reason we choose the crossover region of four steps from the boundary towards the relaxor side is illustrated in Supplementary Fig. S15, where the distribution of predictions for each step or layer is presented. For the first step, the predictions of large U_{re} have very low frequencies, with the largrest density of 60 mJ/cm^3. For the second and third steps, the predictions for the U_{re} move to higher values and the frequencies also increase. Thereafter in the fourth step the tendency is in the opposite direction, i.e., both the U_{re} and corresponding frequencies decrease. This suggests that the fourth or more steps are tending to move into the relaxor region.

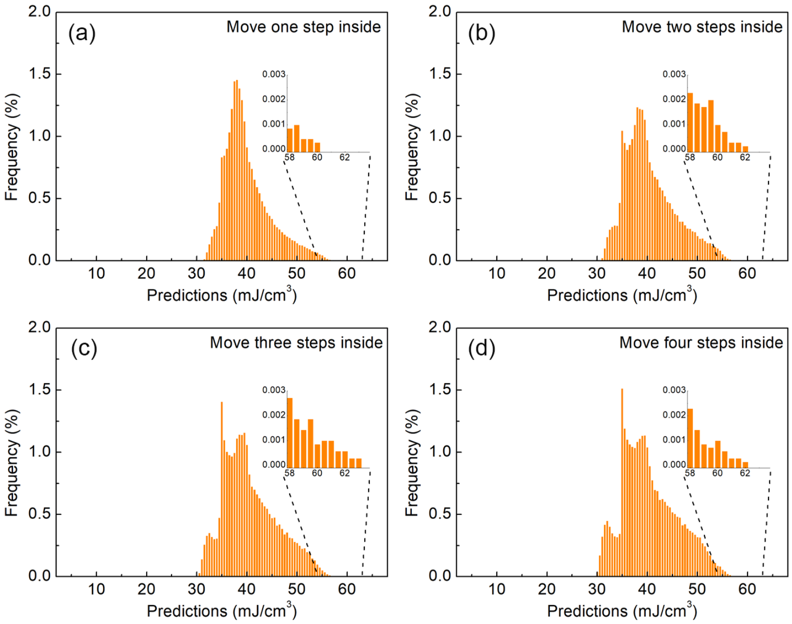

Figure S 14 | **(a)-(d)** correspond to the distribution of predictions for each step, respectively.

* xuedezhen@xjtu.edu.cn
† dingxd@xjtu.edu.cn
‡ txl@lanl.gov
